# Pediatric psoriasis: from immunogenetics to targeted therapies

**DOI:** 10.1016/j.abd.2026.501413

**Published:** 2026-07-03

**Authors:** Adriana Schikiera Martinelli Salathiel, Elisa Nunes Secamilli, Marina Gagheggi Maciel, Juliana Yumi Massuda Serrano, Andrea Fernandes Eloy da Costa França, Renata Ferreira Magalhães

**Affiliations:** a Department of Internal Medicine, Division of Dermatology, Pediatric Dermatology Outpatient Clinic, Hospital das Clínicas, Universidade Estadual de Campinas, Campinas, SP, Brazil; b Department of Internal Medicine, Dermatology Division, Psoriasis Outpatient Clinic, Hospital das Clínicas, Universidade Estadual de Campinas, Campinas, SP, Brazil; c Department of Internal Medicine, Dermatology Division, Pediatric Psoriasis and Dermatology Outpatient Clinic, Hospital das Clínicas, Universidade Estadual de Campinas, Campinas, SP, Brazil

**Keywords:** Psoriasis, Pediatrics, Child health, Comorbidity, Therapeutics, T-Lymphocyte, Antigens, Differentiation

## Abstract

**Background:**

Pediatric psoriasis may result in significant cumulative life course impairment, and there is comparatively less evidence available than for adult psoriasis.

**Objective:**

The aim of this study is to provide an update on the management of pediatric psoriasis, integrating recent immunogenetic and therapeutic advances. It highlights challenges, including clinical heterogeneity, complex differential diagnosis, and limited treatment options, especially in Brazil.

**Methods:**

A narrative review was conducted, including studies published in English, Portuguese, and Spanish between 2009 and 2025, retrieved from the United States National Library of Medicine (PubMed), Cochrane Library, and Scientific Electronic Library Online (SciELO). The following descriptors were used: “psoriasis”, “child health”, “pediatrics”, “therapeutics”, “comorbidity”, and “T-lymphocyte antigen differentiation”.

**Results:**

Pediatric psoriasis most commonly presents as chronic plaque. Differential diagnoses are broad and include atopic dermatitis and autoimmune diseases. Data about comorbidities, particularly cardiovascular risk, are controversial. Although severe cases are less frequent, they are associated with a substantial impact on quality of life. Conventional therapies include topical corticosteroids, phototherapy, and non-targeted systemic agents such as acitretin, methotrexate, and cyclosporine. Biologic therapies have been approved for pediatric use and demonstrate safety profiles and superior efficacy compared to conventional treatments.

**Study limitations:**

Scarcity of pediatric psoriasis guidelines.

**Conclusions:**

Despite advances in understanding adult psoriasis, evidence in pediatric populations remains limited, especially in Brazil. Expanding knowledge in pediatric psoriasis is essential to improve diagnosis, optimize treatment strategies, and increase access to innovative therapies, thereby reducing inflammatory burden and cumulative life course impairment.

## Introdution

Psoriasis is a systemic immune-mediated disease involving skin, nails, and joints. It is associated with metabolic syndrome, cardiovascular disease, obesity, inflammatory bowel disease, and psoriatic arthritis, with a substantial impact on quality of life and mental health. It affects more than 60 million people worldwide,^1^ with a prevalence of 1.3% in Brazil.^2^ The global pediatric prevalence is approximately 1%, with the average age at onset between 7- and 10-years.^3^ It accounts for 4.1% of pediatric dermatoses in Europe and North America.^4^, ^5^

Despite advances derived largely from adult populations, pediatric data, particularly from Brazil and Latin America, remain scarce. This review summarizes immunogenetic advances and therapeutic options in pediatric psoriasis.

## Methods

Given the limited pediatric evidence base, a narrative review was conducted using databases from the United States National Library of Medicine (PubMed), the Scientific Electronic Library Online (SciELO) and Cochrane. Articles published in English, Portuguese, and Spanish between 2009 and 2025 were included. Keywords used: “psoriasis”, “child health”, “pediatrics”, “therapeutics”, comorbidity”, “antigens differentiation T-lymphocyte”.

## Results

### Pathophysiology and immunogenetics

Psoriasis is a polygenic disorder associated with immunological and environmental factors. Well-known triggers include β-hemolytic *Streptococcus* infection, abrupt corticosteroid withdrawal, smoking, lithium, antimalarials, β-blockers, and paradoxically TNF inhibitors.^1^, ^6^

Immunologic process involves innate and adaptive system through activation of T cells, Langerhans cells, and macrophages, dysfunctional keratinocyte differentiation, and impairment of the cutaneous barrier. Autoantigens stimulate dendritic cells within psoriatic plaques to produce Tumor Necrosis Factor-α (TNF-α), Interferon-γ (IFN-γ), Interleukin (IL)-12, and IL-23, thereby directing helper T-lymphocytes (Th) to differentiate into Th1, Th17, and Th22.^7^, ^8^

T-cells that migrate into psoriatic plaques during the inflammatory phase and acquire the capacity to persist either in the dermis (CD4^+^CD69^+^) or in the epidermis (CD8^+^, CD69^+^, CD103^+^) are defined as tissue-resident memory T-cells (TRM). These cells are implicated in lesion reactivation, suggesting that antigen re-exposure, in the presence of TRM and their pro-inflammatory cytokines, are related to disease relapse.^9^, ^10^ Despite limited data, pediatric lesions show distinct signatures: Kim et al. (2020) reported higher CD8 and TNF-α with lower FoxP3 and IL-17A versus adults.^11^ Cordoro et al. (2017) found higher IL-22 and lower IL-17 compared with adult lesions and healthy controls.^12^

### Genetic inheritance

Nearly 20% of patients report a family history of psoriasis, about one hundred susceptibility loci have been implicated, and several designated PSORS. HLA-C*06:02 (PSORS1), located within the major histocompatibility complex at 6p21, shows the strongest association with early onset disease and confers up to a fivefold increase in risk.^13^ It has also been linked to guttate, gestational, and nail psoriasis.^14^ Genome Wide Association Studies (GWAS) have identified different genes related to psoriasis, such as Th1 (IL12B, TYK2) and Th17 (IL23R, IL17R) signaling pathways, innate immunity (NF-κB, TRAF3IP2), and skin barrier function (DEFB4, LCE3B/C), as shown in Table 1.^8^ Over the past two decades, advances in the immunobiology and genetics of psoriasis have enabled highly effective targeted therapies against TNF-α, the IL-23/Th17 axis, and JAK signaling.^15^

**Table 1:** Genes and their functions in antigen presentation, signaling pathways, innate immunity, and skin barrier function.

Signaling / Function	Gene	Action
Antigen presentation	HLA-C*0602	Antigen presentation
	ERAP-1	Modification of peptide binding to MHC-I
Th-1 signaling pathway	IL-12B	IL-12 p40 subunit
	TYK-2	Activation of IL-12
	ZC3H12C	Macrophage activation
	STAT5A/B	IL-12 family signaling pathway
	ILF3	IL-2 expression in T lymphocytes
IL-17 signaling pathway	TYK-2	IL-23 receptor molecules
	JAK2	IL-23 receptor molecules
	STAT3	IL-23 receptor molecules
	SOCS1	Th17 differentiation
	ETS1	Th17 differentiation
	IL17RD	IL-17 receptor
	IL22	Differentiation and proliferation of keratinocytes
	TRAF3IP2	IL-17A/F signaling pathway
	KLF4	Regulation of IL-17A production
Innate immunity	C-REL	NF-κB activation pathway
	TRAF3IP2	NF-κB activation pathway
	CARD14	NF-κB activation pathway
	MICA	Activation of T, NK, NKT cells
	TNFAIP3	NF-κB inhibition pathway
	TNIP1	NF-κB inhibition pathway
	NFKBIA	NF-κB inhibition pathway
	DDX58	IFN pathway and antiviral response
	IFIH1	IFN pathway and antiviral response
Skin barrier function	DEFB4	β-defensin secretion
	LCE3B/C	Epidermal differentiation and hyperproliferation
	GJB2	Connexin 26 and epidermal junctions

Epigenetic mechanisms also contribute to immunopathogenesis by altering gene expression in disease-relevant pathways.^9^, ^15^

### Clinical presentation and diagnosis

Psoriasis has many different clinical phenotypes, whereas the most frequent is chronic plaque, witch symmetric erythematous scaly plaques on elbows, knees, scalp, and lumbosacral area. Other phenotypes include: inverse (involvement of intertriginous areas), genital, erythrodermic (involving > 75% of body surface area with high risk for hypothermia, electrolyte imbalance and cardiac failure), palmoplantar, nail, guttate, and pustular forms.^1^, ^3^ Different psoriasis phenotypes commonly coexist. Beyond skin and nail manifestations, psoriasis also encompasses extracutaneous domains such as arthritis, enthesitis, and dactylitis.^16^

**Fig. 1:**
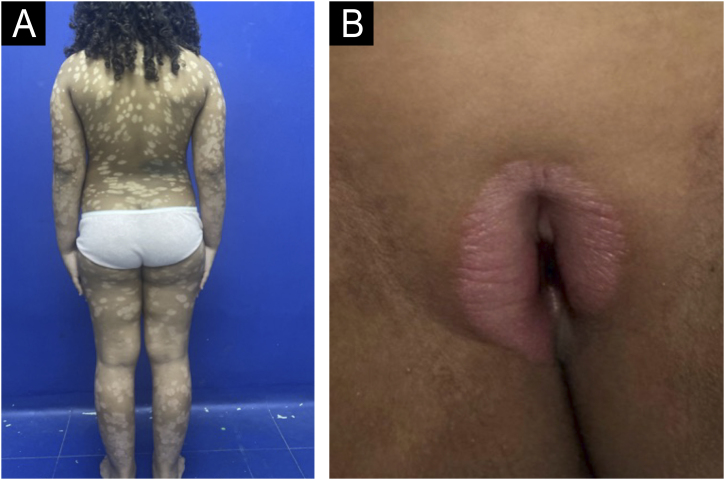
Clinical presentations of psoriasis – (A) disseminated, (B) Genital.

**Fig. 2:**
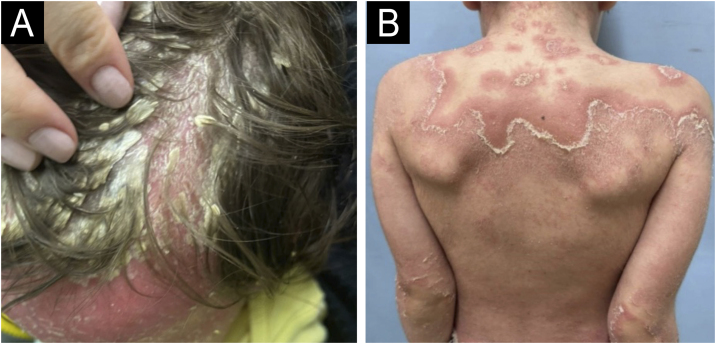
Clinical presentations of psoriasis – (A) Erythrodermic with scalp involvement, (B) Pustular.

**Fig. 3:**
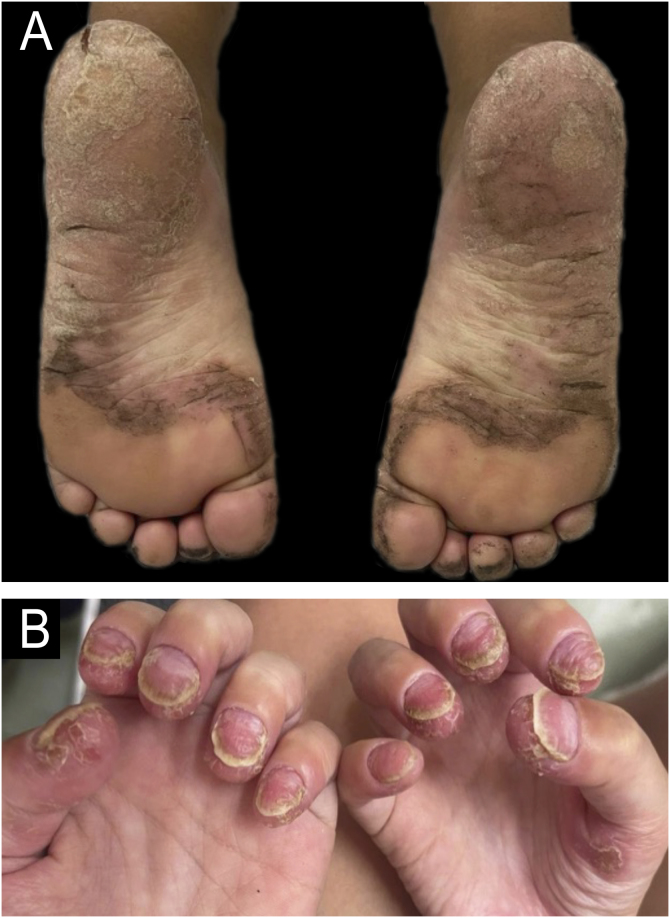
Clinical presentations of psoriasis – (A) Plantar, (B) Nail and palmar.

Pediatric psoriasis presents peculiarities compared to adults. Plaque psoriasis is most common form, accounting 41% of pediatric cases. Often with an abrupt onset, plaques are less scaly and may appear hypopigmented or follicular, with predilection for face, periorificial regions, flexures, anogenital areas, scalp implantation, and umbilicus.^17^ Inverse and diaper psoriasis is the second most common childhood form, followed by guttate, which is characterized by small plaques, often post-infectious.^18^ Nail involvement occurs in 10%–40%, presenting with pitting, onycholysis, subungual hyperkeratosis, and “oil-drop” discoloration.^19^ Pustular psoriasis is rare in children and manifests with sterile pustules on an erythematous base and includes generalized (Von Zumbusch), anular, circinate, exanthematous, and localized palmoplantar variants. Generalized Pustular Psoriasis (GPP) is an acute, severe form requiring urgent care and may be triggered by abrupt systemic corticosteroid withdrawal, hypocalcemia, or infection.^20^ Fig. 1, Fig. 2, Fig. 3 show different clinical presentations of pediatric psoriasis.

**Fig. 4:**
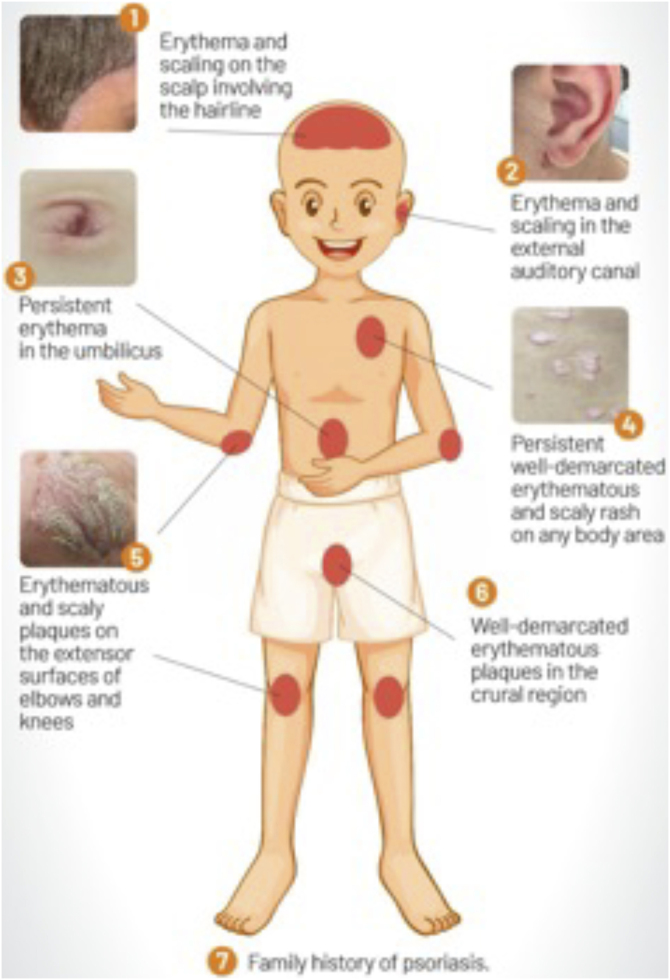
Diagnostic criteria for pediatric psoriasis. Adopted by Burden-Teh et al. 2022.^21^

Burden-Teh et al. (2022) proposed seven diagnostic criteria for pediatric psoriasis (Fig. 4). The presence of two or more positive criteria has 78% of sensitivity.^21^

**Fig. 5:**
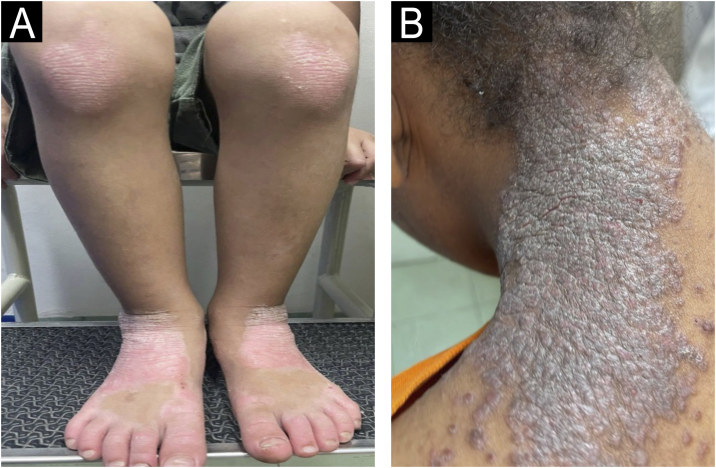
Differential diagnoses for psoriasis – (A) Eritroqueratodermia variabilis, (B) Atopic Dermatitis.

Differential diagnosis in children include: (i) Inflammatory dermatoses, such as atopic, contact or seborreic dermatites, pityriasis rosea, (ii); Infections, such as impetigo, dermatophytosis, candidiasis;^6^ and (iii) Lymphoproliferative disorder, mycosis fungoides and histiocytosis; and (iv) Genodermatoses with erythema and scales ichthyoses and erythrokeratodermia (Fig. 5).

Reports of Psoriasis (PSO) and Atopic Dermatitis (AD) overlap have increased with the advent of biologics, particularly in children and in Asian populations, where clinical, genetic, immunologic, and histopathologic features of both diseases may converge. Asian type AD often shows Th17/Th22 pathway predominance, resembling the erythrodermic forms of PSO or AD. A recent pragmatic classification includes: (i) PSO with AD-like features; (ii) AD with PSO-like features (Asian type AD); (iii) Coexistence “psoriasis dermatitis”; (iv) AD emerging during biologic therapy for PSO; and (v) PSO emerging during biologic therapy for AD.^22^

Autoinflammatory Keratinization Diseases (AIKDs) expand the pediatric psoriasis differential. They are monogenic disorders with innate immune activation, diseases with mixed pathomechanisms of autoinflammation and autoimmunity, superficial dermal and epidermal inflammation, and disordered keratinization. Examples include type V pityriasis rubra pilaris (CARD14), acrodermatitis continua of Hallopeau (AP1S3), and CARD14-associated papulosquamous eruption. Suspect AIKD with early onset, familial clustering, systemic inflammation, or poor response to conventional therapy like methotrexate or acitretin.^23^

**Fig. 6:**
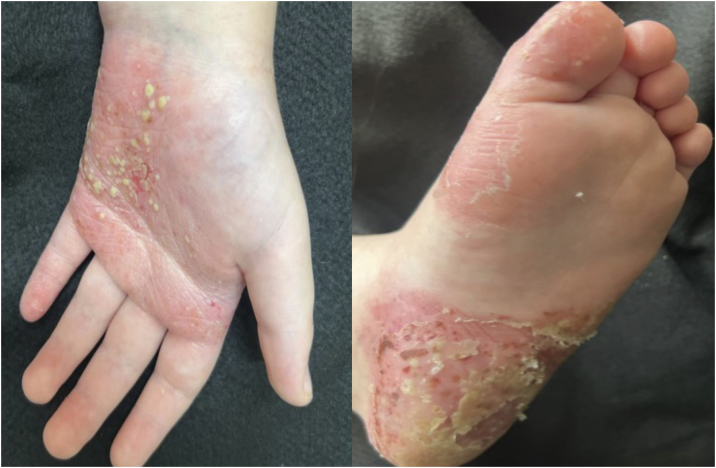
Palmoplantar pustulosis in SAPHO Syndrome (Synovitis, Acne, Pustulosis, Hyperostosis, and Osteitis).

In common syndromic pustular conditions should also be considered, including SAPHO syndrome (synovitis, acne, pustulosis, hyperostosis, and osteitis): Fig. 6, DIRA (deficiency of the IL-1 receptor antagonist)^8^, ^24^ and DITRA (deficiency of the IL-36 receptor antagonist). These entities typically present with very early onset, associated with osteomyelitis, sterile arthritis, and severe systemic inflammation.^8^, ^25^

The diagnosis of psoriasis is primarily clinical, based on recognition of elementary lesions such as erythematous-scaly macules or papules, pustules, palmoplantar hyperkeratosis, and nail dystrophy. In children, attention to typical sites of involvement is essential. Brocq’s methodical curettage supports the diagnosis by demonstrating the *candle-grease* sign (lamellar scaling) and the *Auspitz* sign (pinpoint bleeding after scale removal).^6^ When required, histopathological examination reveals parakeratosis, hyperkeratosis, acanthosis, absence or thinning of the granular layer, elongation of the rete ridges, papillary dermal edema, dilated capillaries, and a perivascular inflammatory infiltrate composed mainly of T-lymphocytes. Aggregates of neutrophils infiltrating the epidermis and dermis may also be observed, forming Munro’s microabscesses or Kogoj’s pustules.^1^

Laboratory and imaging tests are not mandatory for diagnosis but may assist clinical evaluation in the presence of systemic symptoms such as fever, recurrent infections, pain, diarrhea, or in extensive or pustular forms. These include erythrocyte sedimentation rate, C-reactive protein, complete blood count, hepatic and renal function tests, chest X-Ray, or ultrasonography in cases with arthralgia or arthritis, primarily to exclude differential diagnoses or identify associated comorbidities. When genodermatoses or autoinflammatory diseases are suspected, a genetic panel or whole-exome sequencing may be indicated.

### Severity assessment

There are several tools for severity assessment, including the Psoriasis Area and Severity Index (PASI), Body Surface Area (BSA), Physician’s Global Assessment (PGA), and the Dermatology Life Quality Index (DLQI).^26^ For children aged 4- to 16-years, the Children’s Dermatology Life Quality Index (CDLQI) is employed.^27^ Severe psoriasis is defined as PASI > 10, BSA > 10, DLQI > 10,^28^ or pustular psoriasis flare.^14^ In pediatric cases, BSA and CDLQI are more commonly applied.^19^

### Comorbidities and impact on quality of life

The most common comorbidity associated with pediatric psoriasis is Psoriatic Arthritis (PSA), affecting approximately 0.7% of children, with peaks of incidence between 2–3 years and 10–12 years of age. Children with psoriasis also exhibit a two- to four-fold increased prevalence of Crohn’s disease and rheumatoid arthritis compared with those without psoriasis.^18^, ^29^ In adults, the association of psoriasis with metabolic syndrome, cardiovascular disease, and mood disorders is well established, particularly in severe cases.^1^, ^14^ In children, however, the evidence remains conflicting. A meta-analysis including over 40.000 pediatric psoriasis cases demonstrated an association between severe psoriasis and overweight/obesity, as well as an increased risk of metabolic syndrome, diabetes mellitus, hypertension, and ischemic cardiovascular disease, thereby justifying screening in this population.^30^ Other authors argue that in the absence of obesity, cardiovascular risk assessment should follow the pediatric society recommendations according to age group, clinical signs, and symptoms.^29^ Evidence to support systematic screening for non-cardiometabolic comorbidities in children with psoriasis is limited; therefore depending on clinical assessment.^19^

Psoriasis has extensive evidence of quality-of-life impairment, with physical symptoms as itch, skin pain and higher risks of anxiety and depression. Social burden as bullying and stigma, consistently harms their development and is greatest with large body surface area or special site involvement like face, scalp, palms/soles and genital.^31^, ^32^ In Brazilian cohorts, the highest CDLQI means occur in atopic dermatitis and psoriasis.^27^ Early onset, greater severity, and comorbidities contribute to Cumulative Life Course Impairment (CLCI), affecting school and professional performance, relationships and family planning.^32^ Caregivers' impact is measurable by the Family Dermatology Life Quality Index (FDLQI), reflecting the impact of added tasks and costs with appointments and medication management.^18^

### Treatment

Patients with psoriasis should be classified as candidates for topical or systemic therapy, according to disease severity metrics, comorbidities, psoriatic arthritis, patient and family preferences.^33^ General measures include regular emollient use and cardiometabolic risk reduction with physical activity and weight control.

### Topical therapy

The use of emollients and keratolytic agents plays an important role in the management of pediatric dermatoses. Moisturizers and keratolytic agents, such as urea (2%–10%) and salicylic acid (3%–6%), may be incorporated into therapeutic regimens. Urea improves skin barrier function and reduces pruritus, scaling, and hyperkeratosis in patients with psoriasis,^34^ however, evidence regarding the use of urea in children is limited.

Salicylic acid is commonly used as a keratolytic agent, often in combination with topical corticosteroids such as betamethasone dipropionate or mometasone, preferably in children aged ≥12-years. Its application over large body surface areas or at higher concentrations should be avoided due to the risk of salicylism.^14^

Topical corticosteroids are the first-line treatment for pediatric psoriasis. Employed as monotherapy or in combination with vitamin-D analogues. Their use should be avoided on the face, genital and intertriginous areas. Prolonged or extensive use can lead to stretch marks and skin atrophy, in addition to systemic complications, including iatrogenic Cushing's syndrome, growth retardation, diabetes mellitus and high blood pressure.^19^

Topical calcineurin inhibitors, such as tacrolimus and pimecrolimus, are considered safe alternatives for special sites.^35^ Tacrolimus 0.03% is approved for children older than 2-years, and 0.1% for those above 16-years, whereas pimecrolimus 1% is indicated from three months of age.^14^ Vitamin-D analogues are recomended in children more than two years of age, and their combination with topical corticosteroids is considered safe.^6^ See Table 2^14^, ^19^, ^36^ for topical treatment.

**Table 2:** Topical treatment in pediatric psoriasis.

Class / Drug	Main use in pediatric psoriasis	Precautions	Age limits
Low- to mild potency TCS	First-line therapy	Off-label use; avoid FFGD	Any age
Moderate- to high potency TCS	Treatment of thicker plaques	Off-label use; avoid FFGD	≥ 12 years
Tacrolimus 0.03%	Psoriasis involving FFGD	Off-label use	≥ 2 years
Tacrolimus 0.1%	Psoriasis involving FFGD	Off-label use	≥ 16 years
Pimecrolimus 1%	Psoriasis involving FFGD	Off-label use	≥ 3 months
Calcipotriol	Often used in combination with TCS	Avoid application over extensive body surface areas	> 2 years

TCS, Topical Corticosteroid; FFGD, Facial, Flexure, Genital and Diaper area.

### Phototherapy and conventional systemic therapy

Candidates for systemic therapy include patients meeting at least one of the following criteria: 1) BSA or PASI or DLQI > 10%; 2) Involvement of special sites including face, palms and soles, genital area, scalp or nail; 3) Failure of topical therapy; 4) Occurrence of pustular psoriasis flares,^14^ and 5) Comorbidities such as psoriatic arthritis, uveitis, or inflammatory bowel disease.^33^

The therapeutic goal is the achievement of PASI 75, whereas treatment failure is defined as not reaching PASI 50. Patients achieving PASI 50–75 with a DLQI ≤ 5 are considered treatment success.^14^, ^19^ For biologic therapies, the target outcome is PASI 90 or an absolute PASI < 3.^14^

For children with moderate to severe psoriasis, phototherapy is an effective therapeutic option. Narrowband UVB (311–313 nm) is considered safe and is particularly indicated for plaque and guttate psoriasis.^19^

### Acitretin

Acitretin is an oral, non-immunosuppressive retinoid that can be used from six weeks of age, at a dose of 0.1–1 mg/kg/day,^35^ and in young children, capsules may be opened and mixed with milk due to its lipophilicity. Clinical response is generally seen within two months, although pustular psoriasis may improve within 72 -hs. Adverse effects include mucocutaneous xerosis, hyperlipidemia, especially hypertriglyceridemia, and hepatic transaminases elevation. Skeletal abnormality has not been demonstrated at doses up to 1 mg/kg/day, and routine bone imaging is not required unless there are symptoms such as bone pain or impaired mobility. Because acitretin is teratogenic for up to three years after cessation, it is contraindicated in females of childbearing potential. Baseline and periodic monitoring of liver enzymes, lipid profile, and complete blood count is recommended.^19^

### Methotrexate

Methotrexate is an immunosuppressive agent that targets Th1 and Th17 pathways. The recommended dose is 0.2–0.7 mg/kg/week, given orally or subcutaneously. Tablets may be crushed and diluted for easier administration in children. Folic acid supplementation: 1 mg daily except on the methotrexate day, or 5 mg once weekly 24 hours after the dose is recommended to reduce adverse effects such as mucositis, nausea, vomiting, and bone marrow suppression. Less common in children, the adverse effects include pancytopenia, hepatotoxicity, pulmonary toxicity and renal insufficiency. Females of childbearing potential should use contraception and undergo pregnancy testing.^26^ Monitoring includes complete blood count, liver enzymes, and creatinine before and during treatment, as well as serologies for hepatitis B/C and HIV, and chest radiography at baseline. Due to its low cost, effectiveness, and safety profile, methotrexate remains widely used in dermatology.^36^

### Cyclosporine

Cyclosporine inhibits T-lymphocyte activation and suppresses IL-2 and interferon-γ production, thereby blocking inflammatory pathways in psoriasis. It is generally well tolerated and is considered an excellent option for rapid control of severe or pustular pediatric psoriasis.^19^

Available as an oral solution (100 mg/mL), the recommended dose is 2–5 mg/kg/day, divided into two doses, starting at the higher dose and tapering after disease control. Clinical response is often observed within two weeks. Major adverse events include arterial hypertension, nephrotoxicity, hepatotoxicity, and oncogenic potential. Others include hypertrichosis, gingival hyperplasia, hyperlipidemia, hyperuricemia, and hypomagnesemia. Baseline evaluation should include blood pressure, urea, creatinine, electrolytes, complete blood count, lipid profile, liver enzymes, viral serologies, chest radiograph, and pregnancy testing. Blood pressure, complete blood count, lipid profile, and electrolytes should be monitored every two weeks during the first month and monthly thereafter.^26^

### Targeted immunomodulatory therapy for psoriasis

Targeted therapies for psoriasis include biologic agents and small-molecule inhibitors. The first approved class was TNF-α inhibitors (etanercept, infliximab, adalimumab, certolizumab). Subsequent biologics were developed against specific interleukin pathways: IL-12/23 (ustekinumab), IL-17 (ixekizumab, secukinumab, brodalumab, bimekizumab), and IL-23 (risankizumab, tildrakizumab, guselkumab). More recently, the oral TYK2/JAK-pathway inhibitor deucravacitinib was introduced.^37^, ^38^

In a systematic review of therapies for adult plaque psoriasis, Sbidian et al. (2021) reported that IL-17, IL-12/23, IL-23, and TNF-α inhibitors were significantly more effective in achieving PASI 90 compared with conventional therapies and JAK inhibitors, with better results to IL-23 inhibitor.^38^ Sun et al. (2022), using the same study design in pediatric psoriasis, confirmed the efficacy and safety of TNF-α, IL-17, and IL-12/23 inhibitors, although they highlighted limitations due to heterogeneity in study designs.^39^

Biologic agents: adalimumab, etanercept, ixekizumab, secukinumab, and ustekinumab, were approved for pediatric psoriasis according to the U.S. Food and Drug Administration (FDA) and European Medicines Association (EMA).^40^ Guselkumab, a IL-23 inhibitor, represents a recent therapeutic advances in pediatric psoriasis, approved by FDA for children aged ≥ 6-years and ≥ 40 kg, with moderate-to-severe psoriasis and psoriatic arthritis.^41^ In Brazil, therapeutic options for pediatric psoriasis expanded since the approval of TNF-α, IL-12/23, and IL-17 inhibitors for ≥ 6-years,^14^ and most recently guselcumabe for ≥ 12-year-old.^42^ The safety profile of biologics in pediatric patients is comparable to that observed in adults, with mostly mild adverse events such as injection site erythema, upper respiratory tract infections, headache, and náusea.^40^

### TNF-α inhibitors

Etanercept, a TNF-α inhibitor, is the only biologic available in the Brazilian public health system for children ≥6-years with moderate-to-severe psoriasis. It is indicated as second-line therapy when there is inadequate response or contraindication to conventional systemic agents such as methotrexate, cyclosporine, or acitretin.^43^ It is a recombinant fusion protein that blocks TNF-α receptors; its short half-life (2–5 days) and the receptor binding mechanism confer rapid onset and low antigenicity, supporting a favorable safety profile,^44^ although its efficacy is lower than IL inhibitors.^45^

### IL-17 inhibitors

Ixekizumab and secukinumab are anti–IL-17A monoclonal antibodies approved for use in children ≥6-years of age. Both demonstrate high efficacy and safety; however, patients should be monitored for *Candida* infections and for signs of inflammatory bowel disease.^46^

### IL-12/23 inhibitor (ustekinumab)

Ustekinumab is a fully human monoclonal antibody that binds with high affinity and specificity to the p40 subunit shared by IL-12 and IL-23. It is approved for the treatment of psoriasis and psoriatic arthritis in patients ≥6-years of age.

With IL-17 inhibitors, approximately 80%–90% of pediatric patients achieve PASI 75, and more than 70% achieve PASI 90 by week-12. With IL-12/23 inhibition, about 80% achieve PASI 75 and 54% achieve PASI 90 by week-12. By contrast, etanercept shows lower skin clearance rates, with PASI 75 in 56% and PASI 50 in 86% of patients at week-12.^45^

### IL-23 inhibitor (guselcumab)

Guselkumab is a selective IL-23 inhibitor that targets the p19 subunit. A recent phase III randomized placebo-controlled study (PROTOSTAR) enrolled patients aged ≥ 6 to < 18 years with moderate-to-severe plaque psoriasis. Approximately 66% of patients receiving guselcumab achieved PASI 90 compared to 16% of patients receiving placebo at week-16.^41^ Dosing is based on body weight: 1.3 mg/kg (maximum 90 mg) for patients < 70 kg and 100 mg for those ≥ 70 kg, administered subcutaneously at weeks-0 and-4, then every 8-weeks.^41^, ^42^

### Pre-treatment evaluation and vaccination

Before initiating biologic therapy, baseline assessment includes liver enzymes, creatinine, complete blood count, tuberculosis screening, hepatitis B and C serologies, HIV testing, and additional tests guided by clinical history. Vaccination status should be updated prior to initiation of immunosuppressive therapy.^44^ Non-live vaccines may be administered during treatment. Live or attenuated vaccines (BCG, rotavirus, oral polio, yellow fever, MMR, varicella, dengue) should generally be administered 2–4 weeks prior to starting immunosuppressive therapy. If discontinuation of immunosuppressants is required, a period of 4–5 half-lives should be observed before vaccination, and the biologic may be reintroduced 2–4 weeks thereafter.^14^

Table 3 summarizes the recommendations for systemic therapy from medical societies,^14^, ^19^, ^36^, ^47^ and Table 4 presents a guideline for biological therapy regarding pediatric psoriasis.^14^

**Table 3:** Recommendations of medical societies for sistemic treatment for pediatric psoriasis.

Medical Society	Non-biological systemic treatment	Biological therapy ≥6 years old
SBD 2024 [^14^]	First choice	IXQ
	NB UVB and MTX	SCQ
	CYC rescue	USTQ
	ACI special situations	Second line
		ETN
AAD 2020 [^19^]	First choice	1. ETN
	MTX	2. ADLM
	CYC (pustular and Erythrodermic psoriasis)	3. USTQ (> 12 years)
	ACI	
EADV 2020 [^47^]	First choice	ADLM
	MTX	IXQ
	CYC	SCQ
	ACI	Second line
		ETN
		USTQ
SDPL2023 [^36^]	First choice	ADLM
	MTX	ETN
	Second choice or special case:	IXQ
	CYC	SCQ
	ACI	USTQ

SBD, Brazilian Society of Dermatology; AAD American Academy of Dermatology; EADV, European Academy of Dermatology and Venereology; SDPL, Society of Pediatric Dermatology for Latin America; MTX, Methotrexate; CYC, Cycloporin; ACI, Acitretin; IXQ, Ixekizumab; SCQ, Secukinumab; USTQ, Ustekinumab; ETN, Etanercept; ADLM, Adalimumab.

**Table 4:** Biologic therapies for pediatric psoriasis according to Brazilian Society of Dermatology.^14^

Drug	Target	Age (years)	Dose
Etanercept	Anti-TNFα	≥ 6	0.8 mg/kg weekly (maximum 50 mg/week)
Ixekizumab	Anti-IL-17A	≥ 6 and > 50 kg	160 mg at week 0 and 80 mg every 4 weeks.
Secukinumab (indicated for psoriatic arthritis from 2 years of age)	Anti-IL-17A	≥ 6	< 50 kg: 75 mg ≥ 50 kg: 150 mg weeks 0, 1, 2, 3, 4, then every 4 weeks
Ustekinumab (indicated for psoriatic arthritis from 6 years of age)	Anti-IL-12/23	≥ 6	< 60 kg: 0.75 mg/kg ≥ 60 ≤ 100 kg: 45 mg > 100 kg: 90 mg weeks 0, 4, then every 12 weeks

## Conclusion

Most evidence on the pathophysiology and treatment of psoriasis comes from studies in adults and in high-income countries, which limits its applicability to children, particularly in low- and middle-income settings. In Latin America, especially in Brazil, challenges in managing pediatric psoriasis include coexistence with endemic infectious diseases (e.g., tuberculosis, leprosy and leishmaniasis), difficulty in accessing specialized medical care based on treatment guidelines, and restricted access to high-cost medications.^48^

Therefore, it is imperative to expand and disseminate knowledge on pediatric psoriasis, to emphasize differential diagnoses in the era of genetic and immunological discoveries, and to increase the availability of more effective medications for severe cases. Early and adequate treatment is crucial to reduce the impact on quality of life and to prevent cumulative life course impairment in affected children.

## ORCID ID

Elisa Nunes Secamilli: 0000-0001-9036-4200

Marina Gagheggi Maciel: 0000-0001-6077-4209

Juliana Yumi Massuda Serrano: 0009-0000-7748-8583

Andrea Fernandes Eloy da Costa França: 0000-0003-1657-4570

Renata Ferreira Magalhães: 0000-0001-9170-932X

## Declaration on generative AI and AI-assisted technologies in the manuscript preparation process

During the preparation of this work, the authors used Chat GPT to assist in the English translation. After using this tool/service, the authors reviewed and edited the content as necessary and assume full responsibility for the content of the published article.

## Financial support

None declared.

## Authors’ contributions

Andrea Fernandes Eloy da Costa França: Design and planning of the study; collection, analysis, and interpretation of data; critical review of the manuscript.

Renata Ferreira Magalhães: Design and planning of the study; collection, analysis, and interpretation of data; critical review of the manuscript; approval of the final version of the manuscript.

Elisa Nunes Secamilli: Collection, analysis, and interpretation of data; critical review of the manuscript.

Marina Gagheggi Maciel: Collection, analysis, and interpretation of data; critical review of the manuscript.

Adriana Schikiera Martinelli Salathiel: Collection, analysis, and interpretation of data; drafting and editing of the manuscript; critical review of the manuscript.

Juliana Yumi Massuda Serrano: Critical review of the manuscript.

## Research data availability

The entire dataset supporting the results of this study was published in this article.

## Conflicts of interest

Adriana Schikiera Martinelli Salathiel: Support for scientific meetings: Abbvie, Johnson & Johnson, Novartis. Scientific content development: Novartis, Leo Pharma.

Elisa Nunes Secamilli: Support for scientific meetings: Johnson & Johnson, Sanofi, Novartis. Scientific content development: Sanofi, Novartis. Advisory board: Novartis. Speaker: Johnson & Johnson, Sanofi, Novartis, Takeda, Abbvie, Libbs.

Marina Gagheggi Maciel: Support for scientific events: Abbvie, UCB Biopharma. Scientific content development: Leo Pharma.

Juliana Yumi Massuda Serrano: Support for scientific meetings: Abbvie, Johnson & Johnson, Novartis, Sanofi, Eli-Lilly, UCB Biopharma. Clinical research: Eli-Lilly, Novartis. Advisory board: Johnson & Johnson, Novartis, Sanofi, UCB Biopharma. Speaker: Abbvie, Johnson & Johnson, Novartis, UCB Biopharma, Pfizer, Galderma, L'Oreal, Takeda.

Andrea Fernandes Eloy da Costa França: Support for scientific meetings: Abbvie, Pfizer, Lilly, Johnson & Johnson, Theraskin, Sanofi; Clinical research: Lilly, Horizon; Speaker: Abbvie, Pfizer.

Renata Ferreira Magalhães: Support for scientific meetings, clinical research, advisory board and speaker: Abbvie, Johnson & Johnson, Novartis, Lily, UCB Biopharma, Léo Pharma, SunPharma, Bristol Myers Squibb, Boehringer-Ingelheim, Pfizer, La Roche-Posay, Galderma.
